# Facilitating the transition from hospital to home after hip fracture surgery: a qualitative study from the HIP HELPER trial

**DOI:** 10.1186/s12877-024-05390-7

**Published:** 2024-11-15

**Authors:** A Welsh, S Hanson, K Pfeiffer, R Khoury, A Clark, K Grant, P-A Ashford, S Hopewell, PA Logan, M Crotty, ML Costa, SE Lamb, TO Smith, on behalf of the HIP HELPER Study Collaborators, Penny Clifford, Lis Freeman, Rene Gray, James Paget, Yan Cunningham, Sarah Langford, Mark Baxter, Jessica Pawson, Melissa Taylor, Anna Mellows, Kate Lacey, Alex Herring, Diane Williams, Anna Cromie, Gail Menton, Warren Corbett, Helen Jowett, Vishwanath Joshi, Maninderpal Matharu, Maria Baggot, David Barker, Susan Dutton, Opinder Sahota, Katie Sheehan

**Affiliations:** 1 Carnegie School of Sport, Leeds Beckett University, Leeds, UK; 2 Faculty of Medicine and Health Sciences, University of East Anglia, Norwich, UK; 3 Department of Geriatric Rehabilitation, Robert-Bosch-Hospital, Stuttgart, Germany; 4 Nuffield Department of Rheumatology, Orthopaedics and Musculoskeletal Sciences, University of Oxford, Oxford, UK; 5 School of Medicine, University of Nottingham, Nottingham, UK; 6 College of Medicine and Public Health, Flinders University, Adelaide, Australia; 7 College of Medicine and Health Sciences, University of Exeter, Exeter, UK; 8 Warwick Medical School, University of Warwick, Coventry, UK

**Keywords:** Hip fracture, Rehabilitation, Transition, Qualitative

## Abstract

**Background:**

People post-hip fracture have reported experiences of fragmented care and poor discharge planning, therefore improvements in patient flow are required. This study reports the challenges people face during the discharge process and offers potential solutions for improving the transition from hospital to home from the perspectives of patients, carers, and health professionals.

**Methods:**

This was a qualitative study embedded within a multi-centre, feasibility randomised controlled trial (HIP HELPER). We undertook semi-structured interviews with 10 patient-carer dyads (10 people with hip fracture; 10 unpaid carers) and eight health professionals (four physiotherapists, two occupational therapists, one nurse and one physiotherapy researcher) between November 2021 and March 2022. Data were analysed using the principles of Framework Analysis.

**Results:**

Participants identified challenges in the transition from hospital to home post-hip fracture surgery: ineffective communication, disjointed systems, untimely services and ‘*it’s more than just the hip*’. Possible solutions and insights to facilitate this transition included the need for reassurance, collaborative planning, and individualisation.

**Conclusion:**

The transition from hospital to home following hip fracture surgery can be a challenging experience for patients, and for friends and family who support them as carers, making them feel vulnerable, frustrated and uncertain. Enabling a coordinated, collaborative approach to discharge planning and early recovery provision is considered a positive approach to improving NHS care.

**Trial registration:**

ISRCTN13270387. Registered 29th October 2020.

**Supplementary Information:**

The online version contains supplementary material available at 10.1186/s12877-024-05390-7.

## Background

Approximately 76,000 people aged 60 years and older experience a hip fracture in the UK annually [1]. Of these people, 98% undergo surgery [1]. Each year, hip fracture care costs the National Health Service (NHS) more than £869 million in England alone [2].

The provision of post-hospital rehabilitation services across the UK has been described as poor [3]. After hip fracture surgery, people are discharged home or to a nursing/care home or, in more complex cases, receive an additional period of in-patient rehabilitation. Approximately 80% of the 75% of people who live at home before fracture will return home from the hospital [1]. This equates to approximately 45,000 people annually [1]. Three-quarters of patients do not return to their pre-injury level of function by six months [4].

Within 20 days post-surgery, it has been reported that most people receive no or very limited rehabilitation [5–7]. Efforts to support rehabilitation and recovery from hip fracture frequently fall to family members and friends in the role of unpaid carers [8]. Tasks they may assist range from personal activities of daily living (ADL), such as toileting, washing, dressing and cooking, to more complex tasks, such as managing money, shopping and household chores [9]. Caring is heterogeneous in terms of who cares, for example, spouse, children, wider family, and friends, and in what roles people adopt [10].

Previous literature has highlighted some of the challenges carers experience when their friends and family members transition from hospital to home [11, 12]. Fragmented care, which frequently leads to unmet patient needs, adverse events and poor satisfaction with care, especially in patients with multiple chronic conditions, is frequently reported [13–15]. Most studies exploring the care pathway for hip fracture have investigated only one specific component of care, such as care in the surgical ward or standard geriatric consultation [16, 17]. More recently, evaluations of broader health systems have been undertaken [18]. Identification of improvements in patient flow and experience is suggested to improve care and health resource efficiency [18, 19].

We have previously reported the findings from the HIP HELPER feasibility study [10, 20]. The acceptability of an informal carer training programme to support the recovery of people following hip fracture surgery (HIP HELPER) was demonstrated [10]. Caregiver-dyads appreciated contact time with health professionals and the opportunity to develop skills and knowledge for recovery following hip fracture [10]. However, in exploring the perspectives of informal carers supporting people after hip fracture surgery, we observed in the qualitative sub-study that carers felt frustrated, confused, and uncertain during the early, inpatient, recovery phase following hip fracture surgery [21]. As a feasibility study, the trial design demonstrated the successful ability to screen and recruit individuals but was more challenging than anticipated since the research was undertaken during the COVID-19 pandemic [10]. The clinical effectiveness of the informal carer-training intervention was not determined given the nature of this feasibility study and the small sample size to answer the planned research questions [10, 21].

The purpose of this paper was to further report the challenges people face during the discharge process and to offer potential solutions for improving the transition from hospital to home from the perspectives of patients, carers and health professionals.

## Methods

This study was nested within a larger study assessing the feasibility of a pragmatic, multi-centre randomised controlled trial (RCT) of an unpaid carer training programme (HIP HELPER) to support the recovery of people following hip fracture surgery [10]. Caregiver-dyads who were randomised to receive the HIP HELPER intervention were allocated to receive three, 1-hour, one-to-one training sessions. Sessions, delivered by a nurse, a physiotherapist or an occupational therapist, included practical skills for rehabilitation such as transfers and walking, pacing, and stress management techniques, and the provision of the HIP HELPER Caregiver Workbook [10, 20]. This offered information on recovery, exercises, worksheets and goal-setting plans to facilitate a ‘good’ recovery. The intervention was delivered prior to and within the first six weeks after post-hospital discharge, with usual NHS care. The control group received usual NHS care alone following hip fracture surgery. Further details of the mixed methods HIP HELPER study are published elsewhere, in accordance with the Consolidated Standards of Reporting Trials (CONSORT) extension for reporting pilot and feasibility RCTs [10, 20, 22].

Qualitative interviews were used to enable a comprehensive exploration of shared experiences to initially inform a process evaluation [23]. Additional findings sought from the data, irrespective of participant’s intervention group allocation, are reported here. This study was reported in accordance with the Consolidated Criteria for Reporting Qualitative Research (COREQ) guidelines [24].

### Participants

The patient-carer dyad, recruited for the HIP HELPER trial, consisted of an unpaid carer and a person (over the age of 60 years) with a hip fracture. For this qualitative sub-study, dyads were purposively sampled by age, sex, pre-fracture disability status, and hospital location from the main study. An unpaid carer was defined as someone who provided care, assistance, support or supervision in ADLs for at least three hours per week but not on a paid basis. Activities of daily living may include toileting, washing, dressing and eating and/or more complex tasks such as managing money, shopping and household chores [9]. Health professionals sampled for this study were those who delivered the HIP HELPER intervention and were purposively sampled by profession and hospital location. Participants were invited to take part in this qualitative study via telephone call or email. Written informed consent was obtained from all participants.

### Data collection

Between November 2021 and March 2022, semi-structured interviews were conducted with patient-carer dyads. The interviews were held within six weeks of hospital discharge. All interviews were conducted virtually using Microsoft Teams or via telephone by one researcher (AW), who is a white, female, post-doctoral researcher. AW had no role in recruitment to the study nor intervention delivery, thus was not known to participants. The topic guide was developed and piloted with two members of the public and aimed to capture the acceptability of the study and any contextual factors that may have affected fidelity from the perspectives of both dyads and health professionals (Supplementary File 1). Interviews were audio-recorded and transcribed, with all identifying information removed.

### Data analysis

Initially, data was coded deductively against a framework for developing and evaluating complex interventions [23] (findings are reported elsewhere [10, 21]). Open coding (inductive) took place to further explore dyad experiences of hip fracture. The principles of Framework Analysis were applied, in that researchers organised codes into categories that reflected prominent themes within data: challenges experienced in the transition from hospital to home, and possible solutions to improving the pathway, as identified by participants [25, 26].

Data were managed using Excel. AW undertook the coding of transcripts independently; themes and categories were developed through regular discussion among the team (AW, SH, TS, KP). We did not return transcripts to participants for review or correction. This decision was informed by the limited current evidence to support the use of member checks to increase research robustness [27, 28].

## Findings

### Participants

In total, 28 participants were interviewed from five NHS hospitals. Fourteen participant-dyads were invited to be interviewed, of which, 10 participant-dyads consented (10 people with hip fractures and 10 carers). One dyad declined due to other commitments, one did not give a reason and the remaining two dyads did not take part in the interview as the person with hip fracture had been readmitted to hospital. Only one dyad was interviewed separately. The characteristics of the sample are presented in Table 1. The median age of people with hip fractures was 72.5 years (IQR: 69.0. 79.0) and 71.0 years (IQR: 58.0, 77.0) for carers. There was one male and nine females who experienced a hip fracture, and seven male carers and three female carers. Six carers were the spouses of the people with hip fractures, two were adult children (a daughter and a son), and two were described as ‘other’. The median length of interviews was 33 min (range: 27 to 53 min). Eight health professionals participated in an interview (four physiotherapists, two occupational therapists, one nurse and one physiotherapy researcher) across the five sites.

**Table 1:** Characteristics of patient-carer dyad sample

	**Intervention Group** ***N* = 7**	**Control Group** ***N* = 3**	**Overall** ***N* = 10**
**Informal Caregiver**			
Age (years): median (IQR)	71.0 (58.0, 81.0)	71.0 (43.0, 72.0)	71.0 (58.0, 77.0)
Gender: n (%)			
-Male	5 (71.4%)	2 (66.7%)	7 (70.0%)
-Female	2 (28.6%)	1 (33.3%)	3 (30.0%)
Ethnicity: n (%)			
-White British	6 (85.7%)	3 (100%)	9 (90.0%)
-White other	1 (14.3%)	0	1 (10.0%)
Relationship to person with hip fracture: n (%)			
-Spouse	4 (57.1%)	2 (66.7%)	6 (60.0%)
-Daughter/son	1 (14.3%)	1 (33.3%)	2 (20.0%)
-Other	2 (28.6%)	0	2 (20.0%)
Employment: n (%)			
-Not working	6 (85.7%)	3 (100%)	9 (90.0%)
-Part-time work	1 (14.3%)	0	1 (10.0%)
**Person with hip fracture**			
Age (years): median (IQR)	79.0 (70.0, 82.0)	69.0 (68.0, 71.0)	72.5 (69.0, 79.0)
Gender: n (%)			
-Male	1 (14.3%)	0	1 (10.0%)
-Female	6 (85.7%)	3 (100%)	9 (90.0%)
Ethnicity: n (%)			
-White British	7 (100%)	3 (100%)	10 (100%)
Has Cognitive Impairment (based on AMTS category): n (%)	1 (14.3%)	0	1 (10.0%)
AMTS score at consent: median (IQR)	10.0 (9.0, 10.0)	10.0 (10.0, 10.0)	10.0 (9.0, 10.0)
NEADL score at baseline: median (IQR)	20.0 (14.0, 22.0)	10.0 (10.0, 10.0)	17.0 (12.0, 22.0)
Site (n)			
-Site 1	1	1	
-Site 2	1	1	
-Site 3	1	1	
-Site 4	3	-	
-Site 5	1	-	
**Health Professionals**			
Profession			
-Physiotherapist	4		
-Occupational Therapist	2		
-Nurse	1		
-Physiotherapist Researcher	1		
Site (n)			
-Site 1	2		
-Site 2	2		
-Site 3	1		
-Site 4	2		
-Site 5	1		

*Abbreviations*: *AMTS* Abbreviated Mental Test Score, *NEADL* Nottingham Extended Activities of Daily Living Scale

### Themes

**Fig. 1:**
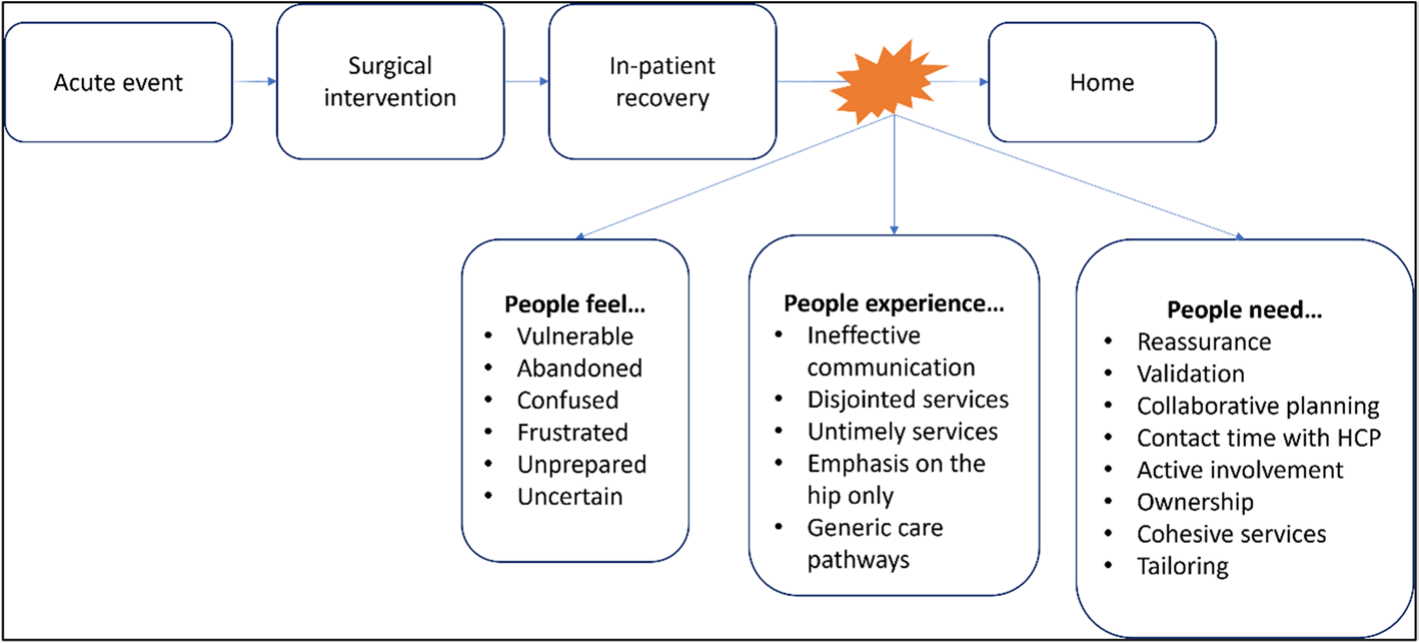
Description of people’s experiences of the transition from hospital to home post-hip fracture

Determined by our analytical framework, we identified the challenges patient-carer dyads and health professionals faced during the transition from hospital to home after hip fracture surgery: ineffective communication, disjointed systems, untimely services and ‘*it’s more than just the hip*’. The findings illustrate what participants experienced and the emotions which these experiences elicited. We also focus on the ‘needs’ of dyads and make inferences of possible solutions to improve the transition home, as suggested by participants: the need for reassurance, collaborative planning, and individualisation. Themes are visually represented in Fig. 1**,** which illustrates experiences, related emotions, and proposed solutions arising from the data.

### Challenges

#### Ineffective communication

Communication between health professionals and dyads was perceived to be ineffective and inefficient. People with hip fracture shared a sense of frustration at managing the lack of communication among staff, themselves and their families.

*“The communication between medical staff and the patient, me, and then my family was almost non-existent. That was extraordinarily difficult to deal with.”*

(Person with Hip Fracture 1, Site 1).

People reported feeling vulnerable at the point of discharge home. Carers suggested they felt uncertain and abandoned, returning home with unanswered questions and sharing their concerns about requiring purposive and clear planning once at home. People with his fracture were anxious they were not receiving the care or services they needed. One carer shared their anxieties around a lack of clear guidance and expectations.

*“I feel like she was just discharged, and we did not know what she was meant to do or how soon or anything”.*

(Carer 1, Female, Site 2).

*‘I feel now though, that I’ve been set adrift’.*

(Person with Hip Fracture 2, Female, Site 4).

Health professionals reported that people post-hip fracture struggled to interpret the information that was being conveyed. They perceived that people were overwhelmed by what was to come, and that dyads were confused by the amount of detailed information shared.

*“You were talking through goals and expectations and what should be happening. You could see that the patient was just, almost a look of, 'Oh my God, is that what's going to happen?' Very overwhelmed.”*

(Occupational Therapist 1, Site 3).

### Disjointed systems

Participants reported experiencing disjointed and dysfunctional systems post-hip fracture. They shared feelings of frustration in dealing with the bureaucracy and felt stressed at the point of navigating and accessing the services needed to support the person with hip fracture. Carers perceived that the systems in place were not responsive to the wider health needs of people with hip fracture, in that there was neglect of ‘smaller details’ (such as wound care). Despite these challenges, carers showed determination in advocating for their loved ones.

*"I’m waiting for the GP to call, to try to get it to another referral, because the wound could flare up. The people from the hospital called me yesterday in the evening to make sure that it was actually happening, and when I said it is not happening, they said that you have to go back and insist. That is what I have been doing this morning and it’s very unpleasant."*

(Carer 2, Female, Site 4)

Health professionals also described the lack of continuity of care, and acknowledged some of the structural and organisational challenges that could hinder the discharge home, such as the changeover of staff. People with hip fracture were also sensitive to this, in that they found it challenging to identify professionals that could consistently address their needs.

*“There should be a bit more patient flow; you know it is very disjointed, you are looked after by completely different nurses and different therapists.”*

(Physiotherapist 1, Site 2).

*“It's quite difficult to figure out which were the experts and they all sort of operate slightly differently”*

(Person with Hip Fracture 1, Site 1).

### Untimely services

People with hip fracture consistently described how out-of-hospital services were not conducive to their rehabilitation journey. For example, some people with hip fracture reported feeling that physiotherapy came much too late. This left them feeling disappointed and frustrated at the absence of services and the time lost waiting for care provision. One person with hip fracture shared a sense of helplessness at their inability to improve their condition.

*“Physio was just non-existent. I’ve been waiting to see a physio and I've wasted like 9 weeks of my life waiting for something and that has probably made it worse. You know, I'm getting worse by the day instead of better now.”*

(Person with Hip Fracture 2, Female, Site 5).

### ‘It’s more than just the hip’

Participants perceived there was a disproportionate emphasis on the hip only, with other health issues that should also be addressed. Although beyond the scope of the present study, to verify if this occurred in practice, people with hip fracture and carers did not describe any health professional contact or discharge planning associated with falls prevention, detecting and/or managing osteoporosis or supporting fragility.

*“They are too focused on the hip, but that's what was happening and everybody else that I've come to contact out here in the community, are not focusing on the fact that Mum’s got other issues that are significant.”*

(Carer 3, Female, Site 3).

There was the perception that the discharge process became a ‘tick box exercise’ and that additional components of their health were negated and could have been addressed more thoroughly.

*“The patient just becomes a tick box exercise. They've got dressing, stockings and stuff, but it is just so much more than that and a lot of it [going home] is psychological.”*

(Carer 3, Female, Site 3).

### Potential solutions

#### Reassurance

Carers sought reassurance from health professionals. This was particularly resonant among first-time carers and reassurance was valued when there was a shift in, or newly assigned, caring responsibilities.

*“I think even if someone had said to me on the day, when mam was being discharged, ‘just totally take it easy for the next two weeks, there's no expectation for you to be able to do anything’. I think that would really help. Told that we were doing the right things and all.”*

(Carer 4, Female, Site 2).

Contact time with health professionals in the home environment was important for carers, as this would allow them to understand their context, manage their assumptions and perceived difficulty of (new) responsibilities.

*“Have someone visit the home environment, not making assumptions that it’s easy because I’m working from home, providing us reassurance.”*

(Carer 4, Female, Site 2).

Furthermore, health professionals suggested that patients were ‘struggling’ post-discharge. This promoted the importance of providing clear information giving and reiterated the necessity of reassurance.

*“A lot of the time, the feedback is that patients are still struggling and still having issues. Whether that's with mobility or pain or whatnot when they're out in the community.”*

(Physiotherapist, Site 2).

Reassurance, signposting and contact time are possible solutions, as identified by both people following hip fracture and carers, to improving the transition from hospital to home, post-hip fracture. An emphasis on this support may elicit improved and effective communication also.

### Collaborative planning

Another potential solution, as alluded to by dyads, is collaborative planning to enable a smooth transition from hospital to home. In this study, people did not feel this was achieved, as they felt unprepared and uncertain about returning home.

Dyads reported a desire to be actively involved in the preparation for activities and felt that they should be included in important decision-making processes. Fostering a sense of ownership and control was considered an important enabler for allowing people to feel included and supported throughout the transition home.

*“They didn’t really ask me. Nobody. Literally, they were trying to send her home and me mam refused because we have nothing [arranged].”*

(Carer 4, Female, Site 2).

A consistent multidisciplinary approach to planning may overcome some of the challenges identified in untimely services and disjointed systems.

### Individualisation

An individualised, holistic approach to planning the discharge home may contribute to addressing the additional needs of people with hip fracture. Carers reported their loved ones had additional needs, and suggested goal-setting (as part of the rehabilitation pathway) needed to encompass other issues.

*“And all these patients, as you know, the elderly have all got other issues and that's why the goal setting is more than the hip.”*

(Carer 2, Female, Site 4).

According to the characteristics of the sample population, the age of people who experienced hip fracture was younger than frequently reported [1]. Hence, a number of people with hip fracture acknowledged that the information and recovery framework applied in current practice did not resonate with them as’younger’ patients, but was designed with older people in mind. Again, this supports the need for an individualised, holistic approach to managing the transition from hospital to home.

*“You know, not all hip replacement patients are elderly either.”*

(Carer 3, Female, Site 3).

*“You know, I'm not a little old lady who sits and knits in the chair and doesn't move about much so that makes a difference, doesn't it?”*

(Person with Hip Fracture, Female, Site 1)

## Discussion

The findings from this study illustrate the challenges dyads and health professionals faced in the transition from hospital to home, after hip fracture surgery. Key challenges included: ineffective communication, disjointed systems, untimely services and negating additional needs, beyond rehabilitating the hip. We were able to extrapolate potential solutions from the dyad’s and health professional’s experiences and propose potential solutions to enhance a smooth transition from hospital to home: reassurance (from health professionals), collaborative planning (of the discharge home) and individualisation (of care).

Communication was reported as ineffective and insufficient. Health professionals in this study suggested that providing information to dyads alone may be overwhelming, given their vulnerability in the hospital after experiencing trauma such as a hip fracture. Previous literature has acknowledged the involvement of both carers and family members in conversations with the multidisciplinary team [27]. Involving all parties (i.e., the patient and their carer) means that the risk of not being able to recall important information is diminished by sharing it with others at the point of contact. The provision of varied and diverse sources of information is also recommended, meaning both patients and carers can access information through their most accessible and preferred means (i.e., paper-based, online, App), and at the time of their choice [27]. Such approaches have previously been demonstrated to increase patient self-management and empowerment [27–29]. Given the dyad’s experiences in the present study, such measures may be desirable within this context.

The provision of care across the transition from hospital to home has been historically challenging, particularly for people from low socioeconomic groups [30]. Previous literature highlights this, with an emphasis on older people [31], people with low health literacy [32] and people who are historically underserved by health services, such as those living in social deprivation [30] and some minority ethnic communities [33]. In the present study, people with hip fracture felt abandoned and anxious they may not be receiving the care or services they require, therefore carers had to advocate for and ‘stand-up’ for the person they support. Carers must also have the knowledge to navigate a perceived complex and ‘disjointed’ care system. While this emphasises the role of carers and the stress that may be placed upon them, it also highlights concerns of further health inequalities for individuals who may not have a carer to act in such a way. Considering such implications provides a strong rationale for health services to overcome these challenges.

There is limited evidence on the perceptions of people who are ‘younger’ and who sustain a hip fracture [34]. The patient perspective was that current care practices for hip fracture surgery recovery across the centres involved in this study were structured towards older people and were not reflective of those who were younger or tailored for those who self-reported to be more physically active. This was concerning the expectations of recovery, the goal-setting trajectory, and the information provision. They felt that health professionals focused on lower recovery potential and expectations, despite being more active and having a greater capacity to improve their condition. From a carer perspective, these individual’s carers are invariably younger with their own social, occupational and family commitments which need to be ‘juggled’. Greater tailoring and flexibility at different ages, as well as co-morbid diseases and social backgrounds, may be required to ensure that future care pathways are adaptable to patient needs rather than assuming that patients are generic, with the same challenges and solutions for recovery.

Both people with hip fracture and carers reported valuing collaborative planning with health professionals during the transition from hospital to home. Effective collaborative planning has been previously reported in discharge approaches among other patient groups, most notably stroke and palliative care [35, 36]. This may be facilitated by a longer hospital stay for many of those patients in comparison to people who sustain a hip fracture [37]. Nonetheless, the values of strong communication, expectation management, delegation of activities and clear timelines to improve patient-carer empowerment are valued across health systems, irrespective of the indication for in-patient stay. Good communication may be seen as a time-consuming activity for health teams. However, based on the findings of this study, such time is valued by both patients and carers for enhancing the hospital-to-home transition. Accordingly, enhanced communication and greater collaborative discharge planning should be considered within hospital services for people following hip fracture surgery.

This study has both strengths and limitations. This study provides unique insights into the perspectives of carers and patients as well as health professionals in the transition from hospital to home after hip fracture surgery. Although patient experiences of hip fracture recovery have been previously reported [21, 38, 39], there remains limited evidence on the views of carers [40]. Therefore, this study advances the understanding of this topic. There are two key limitations to consider. First, all the study participants were ‘White British’ or ‘White Other’ with no carers in full-time work, and 90% not in work at all. Accordingly, the transferability of these results to minority ethnic communities and carers of working age who may have different views of caring, access to health services or experiences with the NHS may be diminished. Further purposive sampling to improve representation would be valuable. Second, the interviews that formed the basis of this qualitative study were undertaken between 2021 and 2022 during the COVID-19 pandemic. Accordingly, this may be regarded as an ‘atypical’ period in health service usage and provision. Reflection on how these findings contrast with current practice, post-pandemic, may be valuable.

## Conclusion

The transition from hospital to home following hip fracture surgery made people feel vulnerable, frustrated and uncertain due to experiences of ineffective communication, disjointed and untimely services, and a lack of individualisation. We have extrapolated several recommendations based on interviews with patient-carer dyads. Employing a coordinated, collaborative and holistic approach to discharge planning is considered by dyads to be a positive step toward improving care. There is also a recommendation to accommodate the needs of ‘younger’ people who experience a hip fracture, with flexible and adaptable services for those who are functionally able. Through such reflections, health professionals could both enhance this transition and promote a stronger, more empowered recovery platform for people following hip fracture surgery.

## Supplementary Information

Supplementary Material 1.

## Data Availability

The datasets used and/or analysed during the current study are available from the corresponding author (AW) upon reasonable request.

## Abbreviations

ADL: Activities of daily living
COREQ: Consolidated criteria for reporting qualitative studies
NHS: National health service
NICE: National institute for health and care excellence
RCT: Randomised controlled trial
