# Supplementary material for: Facilitating the transition from hospital to home after hip fracture surgery: a qualitative study from the HIP HELPER trial
Source: BMC Geriatr. 2024 Nov 15;24:948. doi: 10.1186/s12877-024-05390-7 (PMC11566269; doi:10.1186/s12877-024-05390-7)
Supplement: Supplementary file 1 — Supplementary Material 1. [file 12877_2024_5390_MOESM1_ESM.docx]

Supplementary File: Interview Topic Guide

| **Caregiver Dyads** | | |
| --- | --- | --- |
| **Topic** | **Sample questions** | **Prompts/probes** |
| Overall | - Overall, do share your experiences of being involved with our research? |  |
| The approach and consent process | - Please talk me through how you got involved with the HipHelper research study? | - Could we have dealt with that differently? |
|  | - How clearly was the study explained to you? | - How was this different to what you expected? - For the future, what could we improve about the description of the study? |
| Willingness to be randomised to either group/ participant views of their intervention | - Please talk me through what study treatment you received | - *If required: clarify what HIP HELPER was and what was usual care/non-study intervention* - You were allocated to X group. What did that feel like? |
| The risk of intervention contamination between the groups | - How much did talk to any other patients or caregivers (whilst in hospital) about the intervention? | - What discussions were had between those who received it and did not receive it? |
| **Both groups** | | |
| The acceptability of the inpatient care | - Please talk me through your treatment while you were in the hospital | - What aspects of your care that were lacking in the hospital? - Can you remember which wards you were on? Can you remember being moved to different areas of the hospital? |
|  | - *(Carers)* As X’s carer, what was your impression of the care? | - For both of you, what was helpful and less helpful to your care? |
| **Intervention group only** | | |
| In-patient HIP HELPER programme  Strengths/weaknesses | - How did you find the HIP HELPER programme? | - Please share any specific examples of what you can remember from the programme? - What were the most helpful bits of your HIP HELPER intervention? - What was good about it? - What were the less helpful/worse bits of the HIP HELPER intervention? - For you as the patient/and you as X’s carer? |
|  | - Please tell me what you remember about the manual handling training you had in the hospital? | - How did you get on with that at home? - Was it helpful? If so, how? |
|  | - Please tell me about the phone calls you received? | - Can you remember what you talked about? - Can you give specific examples of what was helpful? - Was there any advice that confused you or you weren't clear about? |
|  | - What were the individualised goals you set in the hospital? | - How did you come up with these? - *(Carer)* By how far were you involved in the goal setting process? - In retrospect, what goal(s) would you alter? |
| **Both groups:** | | |
| On return to your home | - *(If required, state residential status)* Can you tell me who you live with at home? |  |
|  | - What were the first things you wanted to achieve once at home? | - On a 1-10 scale, how confident did you feel when you left the hospital? - Can you tell me why you have chosen that number on the scale? |
|  | - How did you decide who was to be your allocated carer? - *(Carers)* Can you talk me through this decision to be designated as X’s carer? |  |
|  | - *(Long-term carers)* Once at home, how do you feel care-giving role changed? | - Can you tell me about any new caring responsibilities? - How did you organise these? - Did this change the dynamics in their relationship in taking on caring role, if so, how |
|  | - What sort of adaptions did you make at home to enable you to move about independently (where possible)? - What do you think is lacking in the transition from hospital to home? | - Are/were there any other things that may have affected your transfer home? - Are/were there any other things that required you to adapt the advice you were given? |
|  | - How willing were/are you to continue with activities/advice that were suggested to you? - Once at home, by how far did you feel supported by the health care professions you had seen? - *(Carers)* For you as caregiver, how did you get on with helping move X about? - Were there any adaptions that you made to the advice given? | *Probes: transferring, dressing, activities of daily living* |
| What modifications they may recommend to interventions received | - What changes did you find easy to implement and what did you find harder? | - What could we improve? |
|  | - What do we need to modify for any future programmes to support people with a hip replacement and their carer to help you get up and moving and returning to normal life? | - Can you explain why? *Prompt around ADL’s* |
| The ease and convenience of the data collection processes/applicability of the methods and measures used | - As you were part of a trial, we had to collect a lot of measurements. Can you talk me through what these were? | *Prompt:*  *‘Share screen’ of the front page of questionnaires to help with memory recall.* |
|  | - How did you manage with the questionnaires we gave you at the start of the study and at the end in the post? | - Were they easy to complete? - Do you remember them being a problem? - How convenient were they? |
|  | - What about your interview experience today? | - How did you find it? - Is there anything we can do to improve this experience? |
| Summary and end | - We are now ? months since your hip replacement, by how far do you think the HipHelper programme (*or usual care)* has helped with your recovery? | - How far do you think the Hip Helper programme (*or usual care)* has helped you achieve your goals? |
|  | - If we were to run this programme again in the future, is there anything else we should consider? |  |
|  | - How do you think we could better support you and your carer to recover after hip surgery? |  |
|  | - Is anything else you have gained by being involved in this research study |  |
|  | - Is there anything else we haven’t thought of and that you can share with us to improve the study and the care of hip replacement patients and their carers? |  |

| **Healthcare Professionals** | | |
| --- | --- | --- |
| **Topic** | **Sample questions** | **Prompts** |
| Introduction | - Overall, could you share your experiences of being involved with our research? - By how far did you feel COVID-19 impacted the start-up of this study? |  |
|  | - Can you share any thoughts on the recruitment process of this study? | - Why do you think people may have declined to participate? - What are your reflections on the information giving process of study recruitment? - How does HipHelper compare to any other experiences of research study recruitment you may have been involved in? - Do you have suggestions for future conduct or study recruitment? |
| The randomised to either group | - How did you feel about 50% of the patients not receiving the HIP HELPER intervention but getting normal care? | - Did this ‘sit easy’ with you? |
| The acceptability of the inpatient care | - How did the delivery of the HIP HELPER inpatient sessions go? - Was there a decision on professional background? - In your opinion, how did the patients and caregivers get on with it? | - How did you work out who would do what? - Were any modifications made? - Did you feel comfortable teaching all the content? - Did shift working play a part in deciding this? |
| HIP HELPER Telephone Calls | - How did you feel about doing the telephone calls? - In your opinion, how do you think they were helpful for caregivers and patients? - Was it feasible to deliver one call to both members of the dyad? - How did you get on with patients who had cognitive impairment? - Did you make any modifications to the content of the call? - How did you feel at the end of the trial? To what extent did you feel (overall) patients/caregivers needed continued support? | - What are your thoughts on the time points phone calls were delivered – was this sufficient? |
| Training on Intervention | - Did you feel adequately prepared to deliver the inpatient and telephone HIP HELPER interventions? | - Would you recommend any changes to this? - Did you need any additional ‘top up’ or ‘refresher’ training sessions? |
|  | - Overall, can you tell me about the goal setting process with participants? - To what extent do you think the HipHelper programme played a part in achieving these goals? | - How was the caregiver involved? - Were they achievable? - Did goals change at discharge/return home? |
| The risk of intervention contamination between the groups | - Do you think you used the HIP HELPER intervention on control or non-trial patients? - Did other professionals not in the trial use the intervention? | - If either occurred, do you think anything could have been done to avoid this? |
| The ease and convenience of the data collection processes | - As you were part of a trial, we had to collect a lot of measurements. How easy were the intervention data collection logs? | - How convenient were they? - What changes would you recommend if any were needed? |
| Summary and end | - By how far do you think the HipHelper programme (*or usual care)* has helped with patients’ recovery? - By how far do you think HipHelper has reduced burden on care services? - If we were to run this programme again in the future, is there anything else we should consider? - Is anything else you have gained by being involved in this research study? - Is there anything else we haven’t thought of and that you can share with us to improve the study and the care of hip replacement patients and their carers? |  |
